# Lack of association between vascular dementia and Chlamydia pneumoniae infection: a case-control study

**DOI:** 10.1186/1471-2377-4-15

**Published:** 2004-10-12

**Authors:** Soo Chan Carusone, Marek Smieja, William Molloy, Charlie H Goldsmith, Jim Mahony, Max Chernesky, Judy Gnarpe, Tim Standish, Stephanie Smith, Mark Loeb

**Affiliations:** 1 Department of Clinical Epidemiology and Biostatistics, McMaster University, 1200 Main St. W., L8N 3Z5 Hamilton, Canada; 2 Department of Pathology and Molecular Medicine, McMaster University, 1200 Main St. W., L8N 3Z5 Hamilton, Canada; 3 Hamilton Regional Laboratory Medicine Program, 50 Charlton Ave. E., L8N 4A6 Hamilton, Canada; 4 St. Peter's Centre for Studies in Aging, St. Peter's Hospital, 88 Maplewood Ave., L8M 1W9 Hamilton, Canada; 5 Department of Medicine, McMaster University, 1200 Main St. W., L8N 3Z5 Hamilton, Canada; 6 Department of Medical Microbiology and Immunology, University of Alberta, T6G 2H7 Edmonton, Canada

## Abstract

**Background:**

Chronic inflammation appears to play a role in the pathogenesis of vascular dementia. Given the association between *Chlamydia pneumoniae *and stroke, the possibility exists that previous exposure to *C. pneumoniae *may play a role in vascular dementia. The objective of this study was to determine if there was an association between serological evidence of *C. pneumoniae *infection or inflammatory markers with vascular dementia.

**Methods:**

28 case-patients with vascular dementia at a geriatric clinic and 24 caregiver-controls were tested for *C. pneumoniae *IgG and IgA antibodies. The association between vascular dementia and *C. pneumoniae *titres as well as inflammatory markers was estimated by using both conditional logistic regression and stratified logistic regression.

**Results:**

When matched cases were compared to controls, there was no significant difference in elevated *C. pneumoniae *specific IgG antibodies (titre ≥ 1:32), odds ratio [OR] 1.3 (95% confidence intervals [CI] 0.3 to 6.0), p = 0.71, or in elevated *C. pneumoniae *specific IgA antibodies (titre ≥ 1:16), OR 2.0 (95%CI 0.5 to 8.0), p = 0.33 indicative of past or persistent *C. pneumoniae *infection. Similarly, no difference in high IgG or IgA antibody levels (IgG titre ≥ 1:512 or IgA titre ≥ 1:64) between the two groups, indicative of recent *C. pneumoniae *infection, was found, OR 0.4 (95%CI 0.1 to 2.1), p = 0.27. For C-reactive protein (CRP), the mean difference between 18 matched pairs (case – control) was – 3.33 mg/L. There was no significant difference between cases and controls when comparing log transformed values, OR 0.03 (95%CI 0.00 to 2.89), p = 0.13 or comparing CRP values above or below the median, OR 0.8 (95%CI 0.2 to 3.4), p = 0.71. For fibrinogen, the mean difference between pairs (case – control) was -0.07 g/L. There was no statistical difference between cases and controls when comparing log transformed values, OR 0.6 (95%CI 0.0 to 31.2), p = 0.79 or between fibrinogen values above and below the median, OR = 0.5 (95%CI 0.1 to 2.0), p = 0.50.

**Conclusion:**

We found no evidence for a significant association between *C. pneumoniae *infection, inflammatory markers such as CRP and fibrinogen, and vascular dementia.

## Background

Vascular dementia is characterized by a loss of cognitive function and social adaptive functions in individuals with cerebrovascular disease [1,2]. Vascular dementia is the second most common cause of dementia and accounts for 10% to 15% of all cases [3]. The clinical presentation of this illness is variable, depending on the site and extent of the lesion or infarct [2]. The pathogenesis of vascular dementia has not been well defined [1,3]. Chronic inflammation and cytokine dysregulation may play a role [4] similar to that seen in Alzheimer's disease [5].

Recent data from serological and PCR studies support an association between *Chlamydia pneumoniae *and cerebrovascular disease. *C. pneumoniae *has been associated with stroke, transient cerebral ischemia, and atherosclerosis in the middle cerebral artery in both prospective and case-control studies [6-12]. Since stroke is an important precursor to vascular dementia, these data raise the possibility that *C. pneumoniae *infection may also be a risk factor for vascular dementia. To our knowledge, this potential relationship has not previously been assessed. We conducted a pilot case-control study to determine an association between serological evidence of *C. pneumoniae *infection and vascular dementia. We also sought to determine if the inflammatory markers, C-reactive protein (CRP) and fibrinogen were associated with this illness.

## Methods

### Study design

Patients with vascular dementia were enrolled from the Geriatric Clinic at Henderson Hospital, an outpatient clinic affiliated with a tertiary hospital in Hamilton, Ontario. The diagnosis of vascular dementia for participants enrolled was determined in accordance with criteria established by the Neuroepidemiology Branch of the National Institute of Neurological Disorders and Stroke and Association Internationale pour la Recherche et l'Enseignement en Neurosciences (NINDS-AIREN) International workshop [13]. This includes both physical and imaging evidence of strokes, and a temporal relationship between stroke and dementia [3]. Case-patients meeting any of the following criteria were excluded: 1) cognitive impairment due to acute cerebral trauma, hypoxic cerebral damage post cardiac arrest, vitamin deficiency states, central nervous system infection, cerebral neoplasia, significant endocrine or metabolic disease, mental retardation; 2) stroke within the last 6 weeks; 3) patients known, in the past 3 months, to have taken a 7 day or more course of antibiotics with activity against *C. pneumoniae *(erythromycin, clarithromycin, azithromycin, levofloxacin, trovafloxacin, doxycycline, or tetracycline).

The controls for this study were chosen from a list of all caregivers who attended the geriatric clinic at the time of the study, regardless of the diagnosis of their spouse or family member. For each case, one caregiver matched for age (within five years) and sex was selected. Caregivers were excluded if they had a diagnosis that included any of the following: dementia, stroke, or cognitive impairment as determined by a Standardized Mini-Mental Status Examination score of < 27 [14]. Enrolment was from July 1999 to October 2001. All eligible cases and controls who attended the clinic during the study period were approached for consent to participate in the study. Demographic data (age, sex), medical history, and smoking history were collected as well as blood samples for *C. pneumoniae *IgG and IgA antibodies, CRP, and fibrinogen.

This study was approved by the research ethics board at McMaster University. Signed consent was obtained for all participants (proxy consent was utilized for participants considered decisionally impaired).

### Laboratory methods

For *C. pneumoniae *IgG and IgA antibody detection, all sera were titrated at two-fold dilutions from 1:16 to endpoint. Samples were analyzed by microimmunofluorescence (MIF), using a 16 hour incubation of serum and substrate at 4–8°C with the same batch of *C. pneumoniae *IgG/IgM MIF slides (LabSystems OY, Helsinki) (23). To prevent IgG interference, sera used for IgA detection were first treated with goat anti-human IgG antibodies (GullSorb; Gull Laboratories, Salt Lake City, UT, USA). CRP was measured using a high sensitivity automated rate nephelometric immunoassay (Dade Behring high-sensitivity CRP, BNII Nephelometer System, Marburc, DE). Fibrinogen was assayed using an automated STA fibrinogen assay (von Clauss method) on a Roche/Stago (Diagnostica Stago SA).

### Analysis

The presence of elevated antibody levels, indicative of past or persistent *C. pneumoniae *infection, was defined as an IgG titre of 1:32 or greater and IgA of 1:16 or greater [15,16]. High antibody titres to *C. pneumoniae*, suggesting a more recent infection, was defined by IgG titres of 1:512 or greater or IgA titres of 1:64 or greater [16]. Because a skewed distribution was anticipated and a linear relationship with risk was not expected, CRP and fibrinogen were analyzed in two ways: using a log transformation of the values and dichotomizing at the median.

The association between vascular dementia and *C. pneumoniae *titres and inflammatory markers was estimated using a matched analysis. Conditional logistic regression analyses were performed for antibody levels, dichotomized and log transformed CRP, as well as dichotomized and log transformed fibrinogen.

A stratified logistic regression analysis was also conducted for *C. pneumoniae *titres and inflammatory markers (both log transformed and dichotomized at the median), stratifying by age and sex (the following age strata were used: ≤ 70 years, 71–80 years, 81–90 years). All analyses for *C. pneumoniae *titres and inflammatory markers were also performed with adjustment for current smoking status. Data analyses were performed with SPSS version 10 or Egret for Windows version 2.0.3.

The original protocol involved two concurrent case-control studies: one including 30 vascular dementia patients and 30 controls and the other with 30 Alzheimer disease patients and 30 controls. The analysis was to include the additional 30 Alzheimer disease controls in the vascular dementia analysis (giving a 1:2 case:control ratio). Assuming that one third of controls would have elevated *C. pneumoniae *titres, for an alpha of 0.05 and 80% power, matching 30 cases to 60 controls would allow for detection of an odds ratio of 3.8 or higher. As the study proceeded, it became apparent that enrolling the Alzheimer's patients was not feasible. We decided then to limit the analysis to a 1:1 case:control ratio focusing on 30 patients with vascular dementia.

## Results

### Participants

A total of 28 case-patients were enrolled: mean age 76.2 years (minimum to maximum: 56 to 90 years); 18 (64%) were male. Nine of the 28 cases had at least one comorbidity (including angina, coronary heart disease, vascular disease, liver disease and renal disease); 1 case had 3 or more comorbidities. Thirteen of the cases were current smokers.

Twenty of the 28 cases could be matched to caregiver-controls, for a total of 20 case-control pairs. Of these 20 caregiver-controls, 16 were unrelated to a case, and four were spouses of a case. However, none of these four were matched to their spouse. Four additional caregiver-controls were selected for the unmatched analyses, so that data on a total of 24 caregiver-controls was obtained.

Where there was incomplete data on antibody or inflammatory marker levels, those pairs were excluded from matched analyses. Incomplete information on cases and controls occurred when individuals consented to participate in the study and provided medical information but did not attend the out-patient clinic for the required blood collection. All individuals with complete data were included in the unmatched analyses.

### *C. pneumoniae *serology

Univariate analysis of *C. pneumoniae *specific IgG antibodies showed no statistically significant difference in elevated antibody levels between matched case-patients and controls, odds ratio [OR] = 1.3 (95% confidence intervals [CI] 0.3 to 6.0), p = 0.71. When pairs were broken and stratified analyses were performed the difference was not statistically significant, OR = 1.8 (95%CI 0.4 to 8.3), p = 0.46. The analysis was also performed with adjustment for participants' current smoking status, OR = 1.8 (95%CI 0.2 to 12.2), p = 0.57 (see Table 1).

**Table 1:** Comparison of analyses used to assess for associations of *C. pneumoniae *specific serology and vascular dementia

**Variable**	**Conditional**			Stratified*			Adjusted**		
	
	**OR**	**p**	**n^1^**	**OR**	**p**	**n^2^**	**OR**	**p**	**n^2^**
IgG response	1.5	0.66	15	1.8	0.46	48	1.7	0.50	48
IgA response	1.7	0.48	15	2.8	0.13	48	3.0	0.11	48
High titre response	0.4	0.27	15	0.6	0.40	48	0.5	0.34	48

^1 ^Number of pairs included in the analysis

^2 ^Number of individuals included in the analysis

* Stratified on age (≤ 70 years, 71–80 years, 81–90 years) and gender

**Stratified analysis with adjustment for current smoking status

Similarly, no statistically significant difference between elevated *C. pneumoniae *specific IgA antibodies was found between matched pairs, OR = 2.0 (95%CI 0.5 to 8.0), p = 0.33. The stratified analysis produced slightly higher odds ratio estimates, although not statistically significant. For the unadjusted analysis, OR = 2.8 (95%CI 0.7 to 10.4), p = 0.13 and for the adjusted analysis, OR = 2.7 (95%CI 0.5 to 14.2), p = 0.24 (see Table 1).

There was also no statistical difference in high antibody levels between matched pairs, OR = 0.4 (95%CI 0.1 to 2.1), p = 0.27 or in the stratified analysis, OR = 0.6 (95%CI 0.2 to 2.0), p = 0.40 for the unadjusted analysis and OR = 0.5 (95%CI 0.1 to 2.5), p = 0.41 for the adjusted analysis (see Table 1).

### Inflammatory markers

A matched analysis of CRP performed on 18 of the 20 matched pairs (all pairs with complete data) revealed no significant difference between log-transformed values, OR of 0.03 (95%CI 0.00 to 2.89), p = 0.13. Similarly, there was no difference comparing matched cases and controls with CRP values above or below the median, OR = 0.8 (95%CI 0.2 to 3.4), p = 0.71. In the stratified analysis (Table 2), the log transformed CRP variable and the CRP variable dichotomized at the median were not statistically significant in both the unadjusted (OR = 0.5 (95%CI 0.1 to 3.6), p = 0.50 and OR = 2.2 (95%CI 0.7 to 7.2), p = 0.20, respectively) and the adjusted analysis (OR = 0.4 (95%CI 0.04 to 4.7), p = 0.49 and OR = 1.4 (95%CI 0.3 to 6.3), p = 0.64, respectively).

**Table 2:** Comparison of analyses used to assess for associations of inflammatory markers and vascular dementia

**Variable**	**Conditional**			Stratified*			Adjusted**		
	
	**OR**	**p**	**n^1^**	**OR**	**p**	**n^2^**	**OR**	**p**	**n^2^**
LogCRP	0.0	0.13	18	0.5	0.50	49	0.5	0.52	49
CRP	0.8	0.71	18	2.2	0.20	49	2.2	0.19	49
LogFibrinogen	0.6	0.79	18	0.8	0.92	49	0.6	0.83	49
Fibrinogen	0.5	0.33	18	0.6	0.38	49	0.5	0.32	49

^1 ^Number of pairs included in the analysis

^2 ^Number of individuals included in the analysis

* Stratified on age (≤ 70 years, 71–80 years, 81–90 years) and gender

**Stratified analysis with adjustment for current smoking status

A matched analysis of fibrinogen performed on the same 18 pairs revealed no significant difference between the log-transformed values of the two groups, OR = 0.6 (95%CI 0.0 to 31.2), p = 0.79. Similarly, there was no difference when comparing the pairs on fibrinogen values above and below the median, OR = 0.5 (95%CI 0.1 to 2.0), p = 0.33. In the stratified analysis (Table 2), the log transformed fibrinogen variable and the fibrinogen variable dichotomized at the median were not statistically significant in both the unadjusted (OR = 0.8 (95%CI 0.0 to 71.6), p = 0.92 and OR = 0.6 (95%CI 0.2 to 2.0), p = 0.38, respectively) and the adjusted analysis (OR = 0.1 (95%CI 0.0 to 73.9), p = 0.43 and OR = 0.3 (95%CI 0.1 to 1.6), p = 0.17, respectively).

## Discussion

In this case-control study, we found no significant association between elevated or high *C. pneumoniae *specific IgG or IgA antibodies and vascular dementia. To our knowledge, this is the first epidemiologic study to test for an association between vascular dementia and infection with *C. pneumoniae*.

We conducted this study on the basis of evidence linking *C. pneumoniae *to cardiovascular disease and stroke. There is an extensive literature supporting an association between *C. pneumoniae *and atherosclerosis [17-19]. Although the majority of these studies initially focused on coronary heart disease more recent evidence also supports an association with stroke [6-12,20]. However, the clinical importance of this association is uncertain.

Although no significant associations were noted, the relatively small sample size and the odds ratio estimates for elevated IgA and IgG antibodies do not definitively rule out an association. In fact, we powered this study to detect a minimally important association between antibodies and vascular dementia of 3.8. Given that the odds ratio 95% confidence interval of IgG is from 0.3 to 6.0, and 0.5 to 8.0 for IgA, our data do not rule out clinically important associations. The point estimates for elevated IgA and IgG antibodies (2.0 and 1.3, respectively) are similar to the recent meta-analysis odds ratio estimates for coronary heart disease of 1.25 (95% CI 1.03 to 1.53) and 1.15 (95% CI 0.97 to 1.36), respectively [21,22]. In both cases the odds ratio estimate for IgA titres is slightly higher than IgG titres, but not statistically different. The meaning of this difference is uncertain. Danesh et al [21] suggest that these differences are likely due to chance, selection biases, or selective emphasis on particular reports. In contrast, other studies have suggested that IgA titres are more strongly associated with disease outcomes because they are a better indicator of chronic *C. pneumoniae *infection [23,15,10].

Vascular dementia is the second most common cause of dementia, second only to Alzheimer's disease. It was previously believed that most cases of dementia were the outcome of one of these two distinct diseases. However, the clear division between them has recently been challenged. It is now widely believed that vascular risk factors are also associated with Alzheimer's disease and Alzheimer's and vascular dementia may share many common clinical and pathological characteristics [3,24-26]. A number of studies have examined the association between Alzheimer's disease and *C. pneumoniae *infection. In 1998 Balin et al [27] found an extremely high association between the presence of *C. pneumoniae *in post-mortem brain samples and late-onset Alzheimer's disease. However, more recent studies have not repeated these findings [28-31]. A recent randomized controlled clinical trial [16], based on the hypothesis that chronic *C. pneumoniae *infection contributes to Alzheimer's disease, found an improved long-term cognitive state in patients with mild to moderate Alzheimer's disease who had been treated with doxycycline and rifampin. However, the serological data did not suggest that this clinical effect was due to treatment of chronic *C. pneumoniae *infection. One study has looked for *C. pneumoniae *in brain samples of vascular dementia patients. This study, like the later AD studies, did not identify *C. pneumoniae *in any of the brain samples [32]. These results suggest that the presence of *C. pneumoniae *in the brains is not strongly associated with late-onset Alzheimer's disease or vascular dementia.

Inflammatory responses are also known to be associated with cardiovascular disease and have recently been implicated in dementia [33]. Elevated levels of serum C-reactive protein (CRP), a non-specific marker of inflammation, predict cardiovascular disease [34] and dementia [33], and have been associated with stroke patients [35]. Recently, an association between inflammatory markers alpha 1-antichymotrypsin, interleukin 6, and, to a lesser extent, C-reactive protein were associated with an increased risk of dementia [36]. In this study we did not find a significant difference in CRP levels between the cases and controls. This most likely was due to the limited power in the study and the limitations of measuring serum CRP. Although CRP was originally thought to be produced almost exclusively by hepatocytes, CRP is now known to be synthesized in brain cells and upregulated in Alzheimer tissue [37,38]. Consequently, localized increases in CRP may be associated with vascular dementia but not detected with serum measurements.

We found no significant association between increased fibrinogen levels and vascular dementia. Abnormalities of haemostasis are thought to be important in the pathogenesis of cardiovascular disease, ischaemic stroke, and vascular dementia. Within the pathways of coagulation and fibrinolysis, fibrinogen represents an important marker. Elevated levels of fibrinogen are associated with increased risks of cardiovascular disease and ischaemic stroke [39,40] but the results are less conclusive for vascular dementia [41,42]. Lowe and Haverkate [43] believe that because vascular dementia is only one phenotype of the systemic atherothrombosis disease, associations between haemostatic variables and any given phenotype should be interpreted with caution. To show a specific association with a single phenotype, a study would need an extremely large sample size to overcome the overlap in phenotypes and risk factors seen in atherothrombosis.

We acknowledge several limitations of this study. Because of the relatively small sample size, the analyses were adjusted for only a small number of potentially important covariates and the analysis of CRP and fibrinogen was restricted to above and below the median (while quartiles would have been more sensitive). To adjust for variables that were not used as matching criteria and to maximize the data collected a stratified analysis was also done. The additional stratified analysis adjusted for current smoking status. We adjusted for smoking status because there is a known strong association between smoking and *C. pneumoniae *titres; and between smoking and vascular dementia [44]. However, it may also be important to adjust for additional factors that may affect inflammatory markers. We also acknowledge that *C. pneumoniae *serology is an imperfect test of *C. pneumoniae *exposure and chronic infection. First, the high prevalence of *C. pneumoniae *exposure makes it difficult to detect true serological differences between cases and controls. Second, it is unclear what the appropriate serological cut-offs should be for identifying exposure versus chronic infection or recent infections. As a result, different groups have used different criteria making comparisons across studies more difficult. However, the importance of this inconsistency is unclear. In the meta-analysis reported by Danesh et al [21] no significant heterogeneity was found among the studies even though four different cut-off titres were used to determine seropositivity in the microimmunofluorescence assays. An alternative test, that may prove to be more reliable, involves the detection of *C. pneumoniae *DNA in peripheral blood mononuclear cells [45]. Another potential limitation is the choice of controls; because *C. pneumoniae *is infectious an increased exposure in the caregivers could potentially mask a statistically significant association between the patients and controls. There is also evidence that caregivers, because of stress, may have altered immune systems [46] which could interfere with their generation of antibodies and inflammatory markers [47-49].

## Conclusions

In summary, a case-control study of vascular dementia patients suggests that there is no significant association between *C. pneumoniae *antibodies and vascular dementia. We found no evidence for a significant association between systemic inflammatory markers and vascular dementia. While this study can rule out a strong association, larger studies are necessary to determine if a weak association exists.

## Competing interests

The authors declare that they have no competing interests.

## Authors' contributions

ML, MS, WM, CG, JM, and MC conceived and designed the original study. SCC conducted the analysis of data and drafted the manuscript. SS and TS coordinated the study and collected data. JG conducted the serological testing. All authors offered critical input into the manuscript and all have read and approved the final version.

## Pre-publication history

The pre-publication history for this paper can be accessed here:


